# Members of SIAMESE-RELATED Class Inhibitor Proteins of Cyclin-Dependent Kinase Retard G2 Progression and Increase Cell Size in *Arabidopsis thaliana*

**DOI:** 10.3390/life12091356

**Published:** 2022-08-31

**Authors:** Kesuke J. Yamada, Hirotomo Takatsuka, Junya Hirota, Keto Mineta, Yuji Nomoto, Masaki Ito

**Affiliations:** School of Biological Science and Technology, College of Science and Engineering, Kanazawa University, Kakuma-machi, Kanazawa 920-1192, Japan

**Keywords:** cell size control, cell cycle, CDK inhibitor, *Arabidopsis thaliana*, transcriptional regulation, transcription factor, organ growth

## Abstract

Cell size requires strict and flexible control as it significantly impacts plant growth and development. Unveiling the molecular mechanism underlying cell size control would provide fundamental insights into plants’ nature as sessile organisms. Recently, a GRAS family transcription factor SCARECROW-LIKE28 (SCL28) was identified as a determinant of cell size in plants; specifically, SCL28 directly induces a subset of *SIAMESE-RELATED* (*SMR*) family genes encoding plant-specific inhibitors of cyclin-dependent kinases (i.e., *SMR1*, *SMR2*, *SMR6*, *SMR8*, *SMR9*, SMR13, and *SMR14*), thereby slowing down G2 phase progression to provide the time to increase cell volume. Of the *SMR* genes regulated by SCL28, genetic analysis has demonstrated that SMR1, SMR2, and SMR13 cooperatively regulate the cell size downstream of SCL28 in roots and leaves, whereas other SMR members’ contribution remains unexplored. This study shows that in root meristematic cells, SMR9 redundantly participates in cell size control with SMR1, SMR2, and SMR13. Moreover, our cell cycle analysis provides the first experimental evidence that SMR proteins inhibit the G2 progression of proliferating cells. Overall, these findings illuminate the diverse yet overlapping roles of SMR proteins in cell cycle regulation while reinforcing that SMRs are essential downstream effectors of SCL28 to modulate G2 progression and cell size.

## 1. Introduction

Determining the proper cell size is fundamental for multicellular organisms to develop tissues and organs with correct functions and adequate size and structure [1,2]. As in unicellular organisms, such as yeasts, each component cell in developing multicellular structures is believed to possess a mechanism operating the cell autonomously to control its size [1,3]. For proliferating cells, their size is directly affected not only by cell growth but also by division, which typically halves the cell volume by generating two daughters. Cell size is maintained relatively constant by coordinating the rate of cell growth and the interval of each successive cell division during proliferation [3]. 

Unlike extensive studies in yeast and mammalian cells, only a few studies have been known for cell size control in plants, and candidate molecules for cell size sensors have not been proposed until recently. However, earlier studies that employed genetic and pharmacological interference with cell cycle progression revealed that slowing down the cell cycle progression by itself is sufficient to increase cell size in proliferating cells, as is the case for yeast and mammalian cells [4,5,6], illuminating the importance of the negative regulation of the cell cycle in cell size control. As negative regulators of the cell cycle, plants generally have two main classes of inhibitor proteins of cyclin-dependent kinase (CDK): KIP-RELATED PROTEINs (KRP) and SIAMESE-RELATED PROTEINs (SMR) [7]. KRP family proteins show sequence similarity to the *KIP* gene encoding CDK inhibitors in metazoan [4]. Ectopically expressed *KRP*s lead to small, serrated leaves with reduced cell number and increased cell size in *Arabidopsis* [4]. Strong overexpression of *KRP*s interferes with both G1/S and G2/M transition in leaves, resulting in plants with large cells and reduced endoreplication, which is characterized by repeated DNA replication without intervening mitosis and cytokinesis, thus producing cells of higher ploidy levels [7]. 

SMR proteins are specific to plants [8]. The SMR family member was first discovered in *Arabidopsis* mutant, *siamese* (*sim*), which causes ectopic cell division during trichome development [9]. The overexpression of *SMR* genes generally promotes endoreplication, thus producing cells of higher ploidy levels [9,10]. On the contrary, the triple *sim smr1 smr2* mutation decreased the degree of endoreplication in developing leaves [8]. Likewise, the conversion of the endoreplication cycle into the mitotic cell cycle was evidenced in *sim* and *loss of giant cells from organs* (*lgo*)*/smr1* mutants in which single endopolyploid cells, leaf trichomes, and giant cells in sepal epidermis, respectively, become multicellular due to ectopic cell division [9,11,12]. In roots, overaccumulation and reduction of SMRs yield larger and smaller meristematic cells, respectively, indicative of the crucial role of SMRs in cell cycle-dependent cell-size control [13,14]. These studies have gradually uncovered *in planta* function of SMRs, comprising 17 members in *Arabidopsis*, in inhibiting cell cycle progression. Nevertheless, the following questions remain open: (1) What is the fundamental difference between the functions of KRP and SMR family proteins in cell cycle control? Do they regulate different phases of the cell cycle, as often mentioned? (2) Is it possible to consider a member of SMR family proteins as a candidate for cell size sensor, as was shown for KRP4, which is critical for G1/S regulation dependent on cell size? (3) To what extent is each member of the SMR family protein functionally redundant with other members? In other words, is there any function relying specifically on a member or a group of members of the SMR family? 

We recently identified SCL28 as a GRAS-type transcription factor that directly induces a subset of *SMR* family genes to govern cell size in various developing organs in *Arabidopsis* [14]. SCL28 hampers G2 progression to increase cell size, whereas alterations in its expression do not dramatically change organ size due to compensatory changes in total cell number in developing organs [14]. Therefore, we concluded that SCL28 controls the balance between cell size and number, making it possible to modify cell size without affecting overall plant growth [14]. Among the members of the SMR family identified as direct targets of SCL28, SMR1, SMR2, and SMR13 play a crucial role in cell size control downstream of SCL28 in leaves and roots [14]. However, in the absence of conclusive evidence that SCL28-targeted SMRs act as a brake for G2 progression, the recently proposed model whereby SCL28 decelerates G2 progression through induction of *SMR* genes, thereby providing time to increase cell volume, is still not completely established. Furthermore, whether any of the other SCL28-induced SMRs, such as *SMR6, SMR8, SMR9*, and *SMR14*, acts as downstream effectors for SCL28 is also yet to be elucidated [14]. 

In this study, we show that SMR9 plays a role in cell size control at the root meristem in a cooperative manner with SMR1, SMR2, and SMR13, showing a tight genetic interaction with SCL28. Plants with reduced and increased levels of these SMRs displayed acceleration and deceleration of G2 progression in proliferating root meristematic cells, respectively, providing clear evidence that SCL28-induced SMRs function as a molecular brake for G2 progression. Overall, our results demonstrate that SCL28 and SMRs shape the axis for cell size control in plants.

## 2. Materials and Methods

### 2.1. Plant Materials and Growth Conditions

*Arabidopsis thaliana* (ecotype Col-0) was grown on Murashige and Skoog (MS) plates (1x MS salts, 0.5 g/L 2-(N-morpholino) ethanesulfonic acid (MES), 1x MS vitamin solution, 1% sucrose, and 0.4% phytagel (pH 6.3)) under continuous light conditions at 22 °C. The *scl28* and *smr1/2/13* were as previously described [14]. The chemical used was β-estradiol (Wako, Osaka, Japan).

### 2.2. Generation of Mutant and Transgenic Plants

CRISPR-Cas9-mediated genome editing was used to generate *smr9-1* and *smr9-2* mutants [15]. Two regions of the *SMR9* coding sequence, positions 207–226 and 228–247 relative to the translation initiation site, were selected as the target sites of single guide RNAs. The complementary oligonucleotides of these target sites were annealed and ligated to the *Aar*I-treated *pKIR1.1* plasmid with DNA Ligation Kit Mighty Mix (Takara, Shiga, Japan) [15]. The following oligonucleotides were used: 5′-ATTGGAGATGCTGACGTGTCCAC-3′ and 5′-AAACGTGGACACGTCAGCATCTC-3′ for *smr9-1*, and 5 ′-ATTGGCACCGAAGAAGCAAAAGG-3′ and 5′-AAACCCTTTTGCTTCTTCGGTGC-3′ for *smr9-2*. The resulting constructs were introduced into both *smr1/2/13* triple and *scl28 smr1/2/13* quadruple mutants via *Agrobacterium*-mediated transformation using a floral dip method [16]. We selected T2 plants harboring heterozygous mutations at the target sites, from which T3 seeds were obtained. Among the T3 plants, we identified homozygous mutations, *smr9-1* under the *smr1/2/13* background and *smr9-2* under the *scl28 smr1/2/13* background. To identify homozygous *smr9* mutations, genomic fragments of the *SMR9* gene were amplified using the following oligonucleotides: 5′-ACATTTCATGCATTGATCGTC-3′ and 5′-AAATGTTAGGAAGCACCATTC-3′. The resulting PCR products were subjected to DNA sequencing, and T3 plants carrying homozygous *smr9* mutations were isolated.

To generate plants conditionally overexpressing *SMR1*, a binary plasmid, *pER8* [17], was used as a parental plasmid for generating a Gateway destination vector, *pER8:GW-YFP*, by cloning the coding sequence of *YFP* connected to a Gateway conversion cassette. The protein-coding sequence of *SMR1* without the stop codon was amplified by PCR from genomic DNA of wild-type *Arabidopsis* plants using the following primers: 5′-GGGGACAAGTTTGTACAAAAAAGCAGGCTTGATGGATCTTGAATTACTACAA-3′ and 5′-GGGGACCACTTTGTACAAGAAAGCTGGGTTTCTTCGAGAACAATAAGGGTA-3′. The PCR product obtained was cloned into a *pDONR201* vector (Thermo Fisher, Waltham, Scientific, MA, USA) to create a Gateway entry clone of *SMR1*, which was further used for LR reaction with *pER8:GW-YFP* to generate a plasmid in which SMR1-YFP fusion is driven under the control of β-estradiol-inducible promoter. This plasmid was used for the transformation of *Arabidopsis* plants, as described above, in order to obtain plants expressing SMR1-YFP upon β-estradiol treatment.

### 2.3. Microscopy

The area of the leaf palisade cells was measured, as described by Nomoto et al. [14]. In brief, the 1st/2nd leaves of 22-day-old seedlings were fixed in a 9:1 of ethanol and acetic acid solution, cleared with Hoyer’s solution (a mixture of 100 g chloral hydrate, 10 g glycerol, 15 g gum arabic, and 25 ml water), and then subjected to microscopic observations. Images of palisade cells at positions one-fourth and three-fourth from the tip of the leaf were acquired using a differential interference contrast microscope (BX51, Olympus, Tokyo, Japan). The average size of palisade cells in the uppermost layer of palisade tissue was calculated using previously described methods [4].

The measurement of root cell length was conducted according to previous work [14]. To visualize cell outlines, five-day-old roots were stained with 0.05 mg mL^−1^ propidium iodide (PI) and observed under confocal laser scanning microscopy (CLSM) using an inverted fluorescence microscope (Eclipse Ti2, Nikon, Tokyo, Japan) equipped with a confocal scanning unit (A1, Nikon, Tokyo, Japan). The resulting images were processed using ImageJ software to measure the cell length in the root meristem. 

### 2.4. Pulse Labeling with EdU

Pulse labeling with 5-ethynyl-2′-deoxyuridine (EdU) was conducted, as described in ref. [18], with slight modifications. Five-day-old seedlings grown on solid medium were transferred to liquid MS medium containing 20 µM EdU, followed by 15-min incubation. After washing with the liquid MS medium, the seedlings were transferred back into the solid MS medium, and root tips were collected 4 and 8 h after an EdU pulse. Double staining with EdU and DAPI was conducted with a Click-iT Plus EdU Alexa Fluor 647 Imaging Kit, according to the manufacturer’s instructions (Thermo Fisher Scientific, Waltham, MA, USA).

### 2.5. Ploidy Measurement

For ploidy analysis, nuclei were isolated from 1st/2nd leaves of 24-day-old plants, stained using CyStain UV precise P kit (Sysmex, Hyogo, Japan), and then subjected to ploidy measurement using a CyFlow Ploidy Analyzer (Sysmex, Hyogo, Japan). According to a previous study [19], the population of nuclei in each ploidy was estimated.

## 3. Results

### 3.1. SMR9, Together with SMR1, SMR2, and SMR13, Regulates Cell Size in Roots

**Figure 1:**
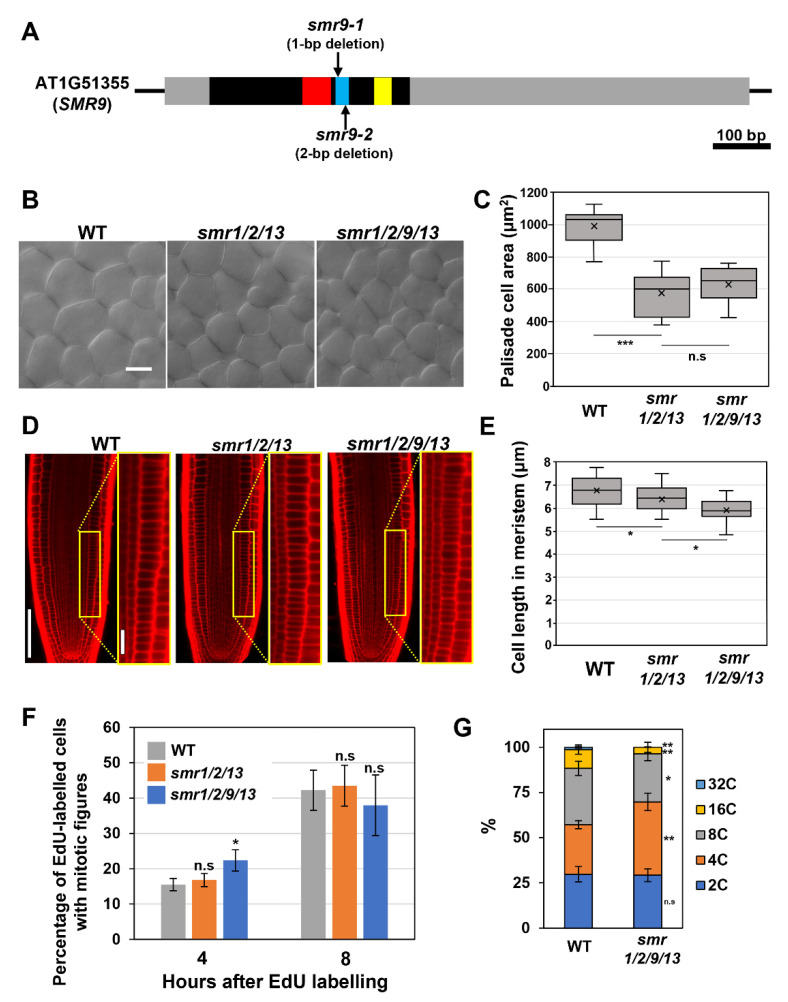
Phenotypic analyses of the *smr1/2/9/13* quadruple mutant. (**A**) Schematic diagram of the *SMR9* gene. The black box, gray boxes, and black lines represent the coding region, UTRs, and intergenic regions, respectively. The red, blue, and yellow boxes represent motif-A, -B, and -C, respectively, which were conserved in SMR proteins. (**B**) Palisade cells from the first leaf pairs of 22-day-old wild-type (WT), *smr1/2/13*, and *smr1/2/9/13* plants. The scale bar represents 20 µm. (**C**) Area of palisade cells in the first leaf pairs from WT, *smr1/2/13*, and *smr1/2/9/13* plants. Data were collected from 10 leaves of different plants. Significant differences comparing with *smr1/2/13* were determined using Student’s *t*-test (*** *p* < 0.001; n.s., not significant). (**D**) Root meristem of six-day-old seedlings of WT, *smr1/2/13*, and *smr1/2/9/13* were subjected to PI staining. Regions surrounded by yellow rectangles are shown at higher magnification in the right panels. The scale bars represent 100 µm (left) and 20 µm (right). (**E**) Length of cortical cells in the root meristem of six-day-old WT, *smr1/2/13*, and *smr1/2/9/13.* Data were collected from more than 15 roots. Significant differences comparing with *smr1/2/13* were determined using Student’s *t*-test (* *p* < 0.05). (**F**) Five-day-old seedlings of WT, *smr1/2/13*, and *smr1/2/9/13* were pulse-labeled with EdU for 15 min, transferred back to MS solid medium, and collected 4 and 8 h after transfer. Root tips were double stained with EdU and DAPI, and meristematic epidermal cells with mitotic figures were counted. The percentages of EdU-positive cells among those showing mitotic figures were calculated, and the data are presented as mean ± SD (*n* = 3). Significant differences from WT were determined using Student’s *t*-test (* *p* < 0.05; n.s., not significant). (**G**) Ploidy analysis of *smr1/2/9/13* quadruple mutants. The first leaf pairs of 22-day-old WT and *smr1/2/9/13* were subjected to flow cytometric analysis to determine ploidy distribution. Data are shown as averages from 10 biological replicates (± SD). Significant differences comparing with WT were determined using the Student’s *t*-test (* *p* < 0.05, ** *p* < 0.01).

Recently, we reported that *SMR1*, *SMR2*, and *SMR13* play a crucial role in cell size control, acting as direct downstream effectors of SCL28 [14]. The *smr1/2/13* triple mutants display reduced cell sizes in both leaves and roots [14]. Notably, some other members of the *SMR* family (i.e., *SMR6*, *SMR8*, *SMR9*, and *SMR14*) have also been identified as direct targets of SCL28, raising a question of their participation in cell size control. Of these *SMR* members, the transcript level of *SMR9* is markedly affected in *scl28* and *SCL28^OE^* [14]. Here, we examined the contribution of SMR9 to cell size control by comparing the *smr1/2/9/13* quadruple mutant with the *smr1/2/13* triple mutant. To obtain *smr1/2/9/13*, mutations were introduced into the *SMR9* gene in the *smr1/2/13* mutant used in our recent work using CRISPR/Cas9 genome-editing system [14]. The phylogenetic analysis conducted by Kumar et al. identified three motifs conserved in SMR family proteins, termed motif-A, -B, and -C, among which our genome-editing targeted motif-B [8]. Notably, the resultant *smr1/2/13 smr9-1^CRISPR^* quadruple mutant (hereafter referred to as *smr1/2/9/13*) carried a 1-bp deletion (C at position 223) in motif-B of the *SMR9* gene, which can lead to the production of truncated proteins lacking intact motif-B and -C (Figure 1A) [8]. Considering that Kumar et al. showed that motif-B included in the SIM protein is essential for its function as a CDK inhibitor and argued that motif-C perhaps plays a role in SIM binding to cyclins [20], it is highly probable that the mutation represents loss-of-function alleles of *SMR9* (Figure 1A).

Although neither *smr1/2/13* triple nor *smr1/2/9/13* quadruple mutants exhibited apparent phenotypes with respect to overall plant growth, leaf palisade cells of both mutants were smaller than those of the wild-type, which shows the role of these SMRs in cell-size control in leaves (Figure 1B,C). More importantly, there was no significant difference in leaf cell size between *smr1/2/13* and *smr1/2/9/13*, suggesting that SMR9 makes little or no contribution to cell-size control in leaves (Figure 1B,C). However, we noticed a further reduction in cell length in the roots when a mutation in *SMR9* was combined with *smr1/2/13* (Figure 1D). To support our observation, the quantitative analysis indicated that the mean cell length in the root meristem of *smr1/2/9/13* was slightly but significantly shorter than that of *smr1/2/13* (Figure 1E). This result suggests that SMR9, in concert with SMR1/2/13, is a crucial regulator of cell size in roots.

SCL28 can exert its inhibitory effect on G2 progression through the induction of *SMR* genes [14], prompting us to test the possibility that *smr1/2/13* and *smr1/2/9/13*, both of which phenocopy *scl28* in terms of cell size, display accelerated G2 progression, as does *scl28*. To this end, we conducted an EdU pulse labeling experiment, which allowed us to compare G2 length between any given genotypes without the need to generate transgenic plants carrying cell cycle markers. During incubation with EdU, cells during the S phase specifically incorporate EdU into replicating DNA. The EdU-labeled root meristematic cells subsequently complete the S phase, pass through the G2 phase, and eventually enter the M phase, exhibiting EdU-positive mitotic figures. Accordingly, in one genotype with a shorter G2 phase, EdU-positive mitotic cells were expected to appear earlier after EdU labeling than in the other (Figure 1F). 

The percentage of EdU-labeled cells among those with mitotic figures was almost the same among all genotypes tested here, 8 h after EdU labeling (Figure 1F). Notably, the ratio was slightly but significantly higher in *smr1/2/9/13* than in wild-type plants 4 h after EdU labeling (Figure 1F), suggesting that G2 progression was accelerated, although subtly, in *smr1/2/9/13*. In support of this view, we found a significant decrease in the ploidy level in leaves of *smr1/2/9/13*, which is generally recognized as a sign of accelerated G2 progression (Figure 1G).

### 3.2. SMR1 Overexpression Significantly Impacts Cell Size and G2 Progression

To better define the impact of SMR proteins on G2 progression, we turned to the overexpression of these proteins. A previous study revealed that the constitutive overexpression of *SMR* genes markedly inhibited overall plant growth [10]. To exclude the possibility that constitutive SMR overproduction affects overall plant growth and secondarily causes aberrant G2 progression, we exploited a β-estradiol-inducible overexpression system. We attempted inducible overexpression of *SMR1*, *SMR2*, *SMR9*, and *SMR13*, of which no stable overexpression lines of *SMR2*, *SMR9*, and *SMR13* were obtained for unknown reasons; therefore, we restricted our attention to the inducible *SMR1* overexpressor (*pER8-SMR1*).

**Figure 2:**
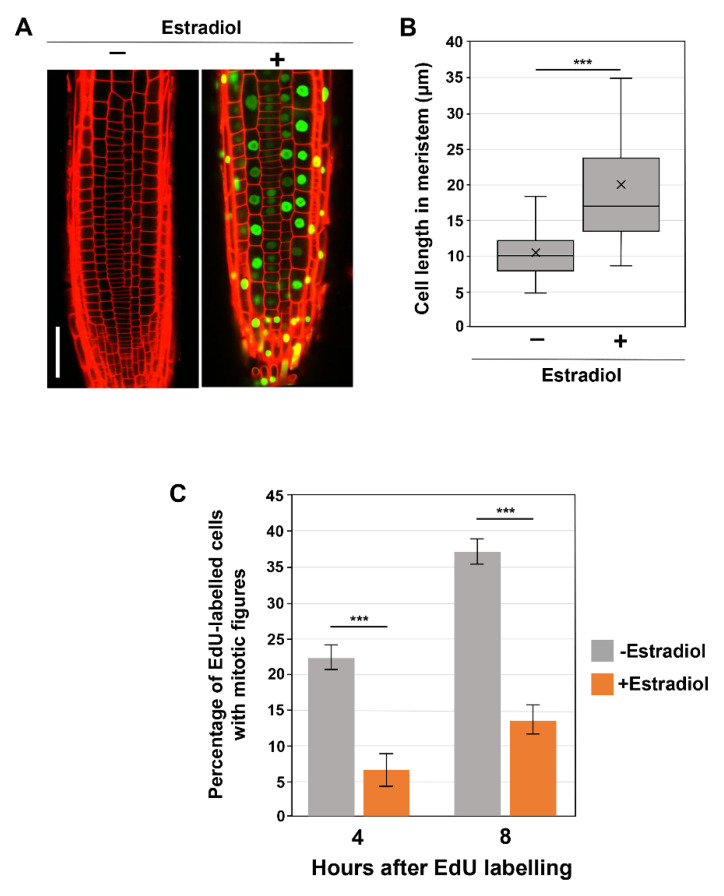
Influence of SMR1 overexpression on cell size and G2 progression. (**A**) Root meristem of *SMR1-YFP* overexpressing lines in the absence or presence of β-estradiol. Ten-day-old seedlings were transferred to the medium containing 10 µM β-estradiol and further grown for 2 days. PI-stained roots were observed under the CLSM. The scale bar represents 20 µm. (**B**) Length of cortical cells in the root meristem of 6-day-old WT, *smr1/2/13*, and *smr1/2/9/13* plants. Data were collected from more than 15 roots. Significant differences compared with plants grown in the absence of β-estradiol (−) were determined using Student’s *t*-test (*** *p* < 0.001). (**C**) Four-day-old seedlings harboring *pER8-SMR1* were grown in the absence or presence of 10 µM β-estradiol for 1 d, pulse-labeled with EdU for 15 min, and transferred back to the medium. Root tips were collected 4 and 8 h after transfer and were double stained with EdU and DAPI. Meristematic epidermal cells with mitotic figures were counted, and the percentages of EdU-positive cells among those showing mitotic figures were calculated. Data are presented as mean ± SD (*n* = 3). Significant differences comparing plants grown in the absence of β-estradiol (−) were determined using the Student’s *t*-test (*** *p* < 0.001).

We observed that the prolonged treatment with β-estradiol for more than 3 days caused strong suppression of the *SMR1-YFP* transgene, probably due to transgene silencing. Thus, *pER8-SMR1* plants were subjected to microscopic observations after 2 days of β-estradiol treatment. As observed in constitutive *SMR1^OE^* lines under the control of the CaMV 35S promoter [13], inducibly overexpressed SMR1 was localized to the nucleus and caused dramatic cell enlargement in root meristems (Figure 2A,B), though no obvious macroscopic phenotype was observed. These results demonstrate the effects of SMR1 reproduced in our inducible system. Thus, we proceeded to the EdU pulse labeling experiment (Figure 2C). When compared to those in the absence of the inducer, the percentages of EdU-positive mitotic cells were dramatically lower in β-estradiol-induced roots at both 4 and 8 h after EdU labeling (Figure 2C), indicating the role of SMR1 in inhibiting G2 progression. These results strengthen the recently proposed idea that SMRs are key direct targets for SCL28 to decelerate G2 progression and thereby increase cell size.

### 3.3. SMR1, SMR2, SMR9, and SMR13 Are the Crucial Downstream Effectors for SCL28

**Figure 3:**
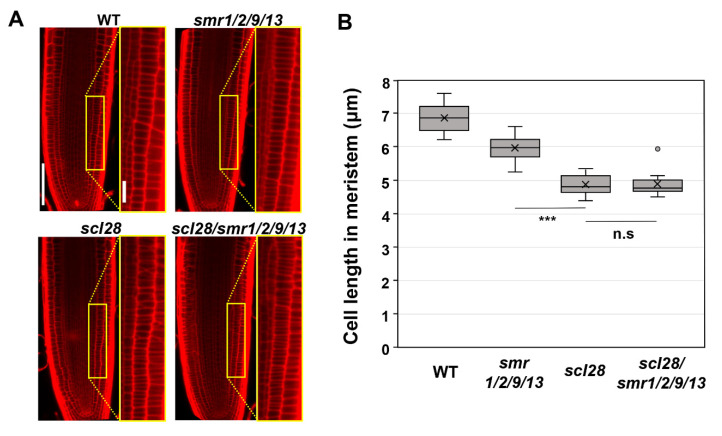
Cell length in root meristem of *scl28*, *smr1/2/9/13*, and *scl28/smr1/2/9/13.* (**A**) Root meristem of six-day-old seedlings of WT, *scl28*, *smr1/2/9/13*, and *scl28/smr1/2/9/13* were subjected to PI staining. Regions surrounded by yellow rectangles are shown at higher magnification in the right panels. The scale bars represent 100 µm (left) and 20 µm (right). (**B**) Length of cortical cells in the root meristem of six-day-old WT, *smr1/2/9/13*, and *scl28*/*smr1/2/9/13* plants. Data were collected from 10 roots. Significant differences from *scl28* were determined using the Student’s *t*-test (*** *p* < 0.001; n.s., not significant).

Finally, we conducted a genetic analysis to determine whether *SMR9* is a bona fide downstream effector of SCL28 in roots, as well as *SMR1*, *SMR2*, and *SMR13* (Figure 3). To this end, we introduced the same CRISPR/Cas9 construct used to generate *smr1/2/9/13* into *scl28/smr1/2/13* quadruple mutants to obtain *scl28/smr1/2/9/13* quintuple mutants. The resulting allele, *smr9-2*, bears a 2-bp deletion mutation in motif-B and thus likely encodes a nonfunctional, truncated SMR9 protein, similar to *smr9-1* (Figure 1A). Both the *scl28* and *smr1/2/9/13* mutants displayed shorter root meristematic cells than the wild-type plants, as reported previously and in this study (Figure 3A,B) [14]. However, the *scl28/smr1/2/9/13* quintuple mutant did not show a further reduction of cell length (Figure 3A,B), suggesting that SCL28 and SMR1/2/9/13 act in the same pathway to regulate cell size at root tips. Overall, our results demonstrate that SCL28 regulates root meristematic cell size at least partly through the direct induction of *SMR1/2/9/13*. Note that the root meristematic cells of *smr1/2/9/13* quadruple mutants were significantly smaller than those of wild-type plants (Figure 1E), but the phenotype was much weaker than that of *scl28* (Figure 3B). This suggests that SCL28 governs cell size not only through *SMR1/2/9/13* but also through other SCL28-regulated *SMR* genes, such as *SMR6, SMR8*, and *SMR14,* and presumably non-*SMR* targets as well [14].

## 4. Discussion

According to previous studies [8,9,11], SMR proteins undoubtedly function as CDK inhibitors in cell cycle progression. Nonetheless, the cell cycle stage in which each SMR acts remains unclear. Several studies have shown that some members of SMR proteins play inhibitory roles in G2 progression. First, biochemical studies have revealed that SIM inhibits the activity of G2/M-driving CDK, namely CDKB1 in vitro [8]. Second, in the absence of *SIM* and *SMR1*, leaf trichomes and giant cells in the sepal epidermis undergo extra cell division [9,11,12], probably due to the failure in the cessation of the mitotic cell cycle at the G2 phase. Third, *sim smr1/2 triple* mutants and *SMR* overexpressors show lower and higher ploidy levels [8,10], which can be interpreted as accelerated and delayed G2 progression, respectively. However, given that the ploidy level is generally associated with the developmental status in *Arabidopsis*, it cannot be denied that the deficiency and overproduction of SMR proteins alter the progression of cell cycle stages other than the G2 phase to affect plant growth, ultimately leading to lower and higher ploidy levels, respectively. Thus, this study’s results provide the first direct evidence that SMR proteins have a molecular function in delaying G2 progression in proliferating cells (Figure 2C). Notably, this does not rule out the possibility that SMRs (SMR1/2/9/13 and other members) are also engaged in the G1/S progression. In support of this view, a recent study showed that SMR4 retards G1 progression in the cell cycle of stomatal precursor cells [21]. Considering that SMR8 modulates cell cycle progression in stomatal precursor cells cooperatively with SMR4, it is reasonable to assume that SMR8 is also engaged in delaying G1 progression in stomata lineages [21]. Meanwhile, our ChIP-seq analysis using whole seedlings included *SMR8* in the list of direct targets of SCL28 [14], leading us to consider that SMR8 may inhibit G2/M progression as a part of the SCL28-governed transcriptional network. In light of these findings, it seems likely that the SMR family might comprise those working in the G2/M, those in the G1/S, and those possibly in both and that each member could alter the cell cycle stage it acts, either in a cell type-specific or developmental context-dependent manner. 

Another important question that needs to be addressed in future research is why plants possess numerous SMRs, each with distinct and overlapping functions in cell cycle progression. One possible explanation is that deploying different SMRs for each cell lineage, tissue, and organ, as well as for each cell cycle stage, might be required to accomplish flexible and precise control of the cell cycle. To support this idea, there are many examples illustrating highly divergent but somewhat overlapping functions of 17 *SMR* genes encoded in the *Arabidopsis* genome: (1) The *sim smr1/2* triple mutants exhibit a lower ploidy level in leaves than any of the *sim*, *smr1*, or *smr2* single mutants [8]; (2) Our recent and present studies illuminate the redundant role of SMR1, SMR2, SMR9, and SMR13 in cell size control at the root tip [14]; (3) As mentioned above, SMR4 and SMR8 act together to inhibit cell division in stomata lineages [21]; (4) A recent study revealed the possible role of SMR6 and SMR11 in lateral root initiation [22]; and (5) SMR4, SMR5, and SMR7 are highly upregulated in response to DNA damage [10]. Despite these remarkable advances, it is yet to be fully explored which SMRs are involved in which processes during plant morphogenesis, organ growth, and stress response. To tackle this issue, in addition to exploring other SMR members that have not yet gained attention, large-scale combinatorial phenotyping, ranging from lower- to higher-order *smr* mutants, will be essential. Furthermore, it will be of particular importance in the future to unveil the functional relationship between SMRs and KRPs, another class of CDK inhibitors in plants. To our knowledge, there have been no studies focusing on how different and similar KRPs and SMRs are in terms of the control of cell cycle and cell size. Nonetheless, it is reasonable to assume that as-yet-unidentified cooperative and/or antagonistic interactions between KRPs and SMRs exist in proliferating cells since some of the KRP and SMR gene families appear to overlap in their expression domains of meristematic tissues in roots and shoots [10,23,24]. 

In this study, we showed that a suite of *SMR* genes are critical targets of SCL28 acting as a regulator of cell size in *Arabidopsis*. This raised the possibility that not only SCL28 itself but also SMRs, may be a candidate for molecule sensing cell size and regulating the cell cycle in plant cells. Recent reports in mammalian cells have identified retinoblastoma (RB) protein as a cell size sensor, which is known to act as a negative cell cycle regulator by binding to E2F transcription factor and inhibiting G1/S cell cycle transition [25]. RB, synthesized during S/G2/M, is transmitted into daughter cells at division and subsequently diluted by the increase in cell volume during G1 until its concentration meets the threshold when cells are allowed to initiate DNA replication. Consistently, the size in mammalian cells is strongly affected by RB expression levels both in vivo and in cells in tissue culture. A similar dilution mechanism has been proposed for cell size control in budding yeast, in which dilution of Whi5, with a role equivalent to RB for negative regulation of cell cycle, is critical for cell size-dependent G1/S transition [26]. In contrast, little is known about the factor(s) that coordinate(s) cell cycle progression with cell growth in plant cells. In the inhibitor dilution model, which has been proposed for cell size control in yeast and mammalian cells, the potential cell size sensor should have the ability to inhibit the cell cycle at a specific transition and decrease in its concentration as cells progress cell cycle and increase their size due to cell growth [3,27]. RETINOBLASTOMA-RELATED1 (RBR1), a homolog of RB in *Arabidopsis*, did not show dilution during cell growth in shoot apical meristem (SAM) cells and, thus, was excluded from the candidate protein for cell size sensors [28]. A recent report showed a member of KRP family proteins, KRP4, as a cell size sensor in SAM cells in *Arabidopsis* [28]. In the proposed model, the dilution of KRP4 proteins is a basis of size-sensing mechanisms acting at G1/S transition in SAM cells. KRP4 proteins synthesized in G2 in mother cells are inherited into daughter cells at division and diluted during cell growth until its concentration reaches a threshold when the inhibition of KRP4 is sufficiently low and G1/S transition is permitted [28]. By contrast, the factor that senses cell size at the G2 phase and triggers entry into the M phase is yet to be identified, although CDKB1, its interacting partner, or downstream effectors have been listed as candidates [29]. Given the phenotype of *scl28* and *smr* multiple mutants shown in this study (Figure 3), SCL28 and/or a defined set of SMRs may also be potential candidates for cell size sensors; how SCL28 and SMR proteins behave during G2/M progression will greatly help test this intriguing hypothesis.

## Data Availability

Data are contained within the article.
